# TRENDS IN SUPPORTS FOR FAMILY CAREGIVERS OF OLDER ADULTS THROUGH MEDICAID HOME AND COMMUNITY-BASED SERVICES IN THE US

**DOI:** 10.1093/geroni/igad104.0177

**Published:** 2023-12-21

**Authors:** Katherine Miller, Elise Parrish, Johanna Thunell

**Affiliations:** Johns Hopkins Bloomberg School of Public Health, Baltimore, Maryland, United States; University of Pennsylvania, Philadelphia, Pennsylvania, United States; University of Southern California, Los Angeles, California, United States

## Abstract

The aging population in the United States, combined with the dominant preference of older adults to remain in the home and community, has increased the demand for unpaid (family) caregivers - a role often undertaken by spouses. Despite known adverse effects of caregiving on health and economic well-being, few systematic policies financially supporting caregivers exist. Medicaid Home- and Community-Based Services (HCBS) 1915(c) waivers are a primary mechanism for paying family caregivers. Our objective is to systematically document and describe supports for family caregivers through Medicaid HCBS 1915(c) waivers. To accomplish our objective, we scrape data directly from approved waivers (2010-2019) serving adults aged 65+ including, but not limited to: approval date, services provided, limitations of services, allowed payments to relatives and legally authorized representatives, and the unique number of enrollees to be served by each waiver. We then describe training/education and payments available to family caregivers. Approximately 62 waivers across 15 states provide training and education to family caregivers. Waivers across 41 states (n=241) allow for payments to legally authorized representatives through 1915(c) waivers. However, upon examining the restrictions of these waivers, 67.6% of these waivers effectively exclude spouses from receiving payments. Despite growing reliance on family caregivers, supports are not widespread and existing supports reach a limited group of family caregivers. This research further contributes to Medicaid research by creating a database of waiver services; thereby contributing to our understanding to the depth and generosity of HCBS, which can be systematically updated as new waivers are approved.

